# REAL-WORLD EFFECTIVENESS OF HIGH-DOSE DUAL THERAPY VS OPTIMIZED CLARITHROMYCIN TRIPLE THERAPY FOR *HELICOBACTER PYLORI*: A PROSPECTIVE COHORT STUDY WITH TEST-OF-CURE SENSITIVITY ANALYSES

**DOI:** 10.1590/S0004-2803.24612026-014

**Published:** 2026-08-14

**Authors:** Alejandro UGARTE LASTRA, David E. ADVINCULA SOLIS, Harold RAMOS MAMANI, Hugo Guillermo CEDRÓN CHENG, Álvaro BELLIDO-CAPARO, Dora MONTEZUMA CALVO, Eduardo VESCO MONTEAGUDO, Günther POPPELE MOLINA, Juan Antonio CHIRINOS VEGA, Jorge ESPINOZA-RÍOS, Juan Sebastián FRÍAS-ORDOÑEZ, Víctor AGUILAR SÁNCHEZ, José Luis PINTO VALDIVIA, Jorge HUERTA MERCADO TENORIO

**Affiliations:** 1 Universidad Peruana Cayetano Heredia, Faculty of Medicine Alberto Hurtado, Lima, Peru.; 2 Clínica Anglo Americana, Department of Gastroenterology, Lima, Peru.; 3 Clínica San Felipe, Department of Gastroenterology, Lima, Peru.; 4 International Hospital of Colombia, Division of Gastroenterology, Bucaramanga, Colombia.

**Keywords:** Helicobacter pylori, eradication therapy, drug therapy, combination, treatment outcome, prospective studies, comparative effectiveness, Helicobacter pylori, terapia de erradicação, terapia combinada com medicamentos, resultado do tratamento, estudos prospectivos, efetividade comparativa

## Abstract

**Background:**

Helicobacter pylori eradication rates have declined globally due to rising antibiotic resistance, challenging the effectiveness of standard triple therapy. High-dose dual therapy has emerged as a simplified alternative supported by pharmacodynamic rationale. We aimed to compare the real-world effectiveness, adherence, and safety of high-dose dual therapy versus optimized clarithromycin-based triple therapy in routine gastroenterology practice.

**Methods:**

We conducted a prospective, multicenter observational cohort study in adult patients with confirmed H. pylori infection treated with either high-dose dual therapy (amoxicillin 1 g three times daily plus esomeprazole 40 mg three times daily for 14 days) or optimized clarithromycin-based triple therapy (amoxicillin 1 g twice daily, clarithromycin 500 mg twice daily, and esomeprazole 40 mg twice daily for 14 days) according to physician decision. Eradication was assessed by carbon-14 urea breath test ≥4 weeks after therapy. Comparative effectiveness was estimated with effect measures, 95% confidence intervals (CI), and predefined sensitivity analyses for incomplete test-of-cure follow-up.

**Results:**

Among 218 treated patients, 104 completed post-treatment breath testing. Eradication rates were 80.4% with high-dose dual therapy and 81.3% with triple therapy (absolute difference −0.9%; 95%CI −16.3 to 14.5; *P*=0.91). Moderate-to-high adherence was observed in 51.5% vs 67.6%, respectively (*P*=0.19). Adverse events were more frequent with high-dose dual therapy (70.8% vs 58.9%), although the difference was not statistically significant. Mainly gastrointestinal intolerance and taste disturbance. Sensitivity analyses across plausible missing-outcome scenarios showed stable comparative results.

**Conclusion:**

In prospective real-world practice, high-dose dual therapy achieved eradication effectiveness comparable to optimized triple therapy, with favorable tolerability. These findings support high-dose dual therapy as a pragmatic and clinically attractive alternative in settings with evolving resistance and adherence constraints.

## HIGHLIGHTS

• High-dose dual therapy and optimized clarithromycin triple therapy showed comparable eradication rates (~80%).

• Neither regimen achieved the ≥90% target eradication threshold in real-world practice.

• Adherence was lower with dual therapy, likely due to higher dosing frequency.

• Adverse events were common but mostly mild in both groups.

• High-dose dual therapy represents a pragmatic, stewardship-aligned alternative in high-resistance settings.

## RESUMO

**Contexto:**

As taxas de erradicação do *Helicobacter pylori* têm diminuído globalmente devido ao aumento da resistência aos antibióticos, comprometendo a eficácia da terapia tripla padrão. A terapia dupla em altas doses surgiu como uma alternativa simplificada, com base em racional farmacodinâmico. Nosso objetivo foi comparar a efetividade em mundo real, a adesão e a segurança da terapia dupla em altas doses versus a terapia tripla otimizada baseada em claritromicina na prática rotineira de gastroenterologia.

**Métodos:**

Conduzimos um estudo de coorte observacional prospectivo, multicêntrico, em pacientes adultos com infecção por *H. pylori* confirmada, tratados com terapia dupla em alta dose (amoxicilina 1 g três vezes ao dia mais esomeprazol 40 mg três vezes ao dia por 14 dias) ou terapia tripla otimizada baseada em claritromicina (amoxicilina 1 g duas vezes ao dia, claritromicina 500 mg duas vezes ao dia e esomeprazol 40 mg duas vezes ao dia por 14 dias), de acordo com a decisão do médico assistente. A erradicação foi avaliada por teste respiratório com ureia marcada com carbono-14 ≥4 semanas após o tratamento. A efetividade comparativa foi estimada por medidas de efeito, intervalos de confiança de 95% (IC) e análises de sensibilidade pré-definidas para seguimento incompleto do teste de cura.

**Resultados:**

Entre 218 pacientes tratados, 104 completaram o teste respiratório pós-tratamento. As taxas de erradicação foram de 80,4% com terapia dupla em alta dose e 81,3% com terapia tripla (diferença absoluta −0,9%; IC95% −16,3 a 14,5; *P*=0,91). Adesão moderada a alta foi observada em 51,5% versus 67,6%, respectivamente (*P*=0,19). Eventos adversos foram mais frequentes com a terapia dupla em alta dose (70,8% vs 58,9%), embora a diferença não tenha sido estatisticamente significativa. Predominaram intolerância gastrointestinal e alteração do paladar. Análises de sensibilidade em cenários plausíveis de desfechos ausentes mostraram resultados comparativos estáveis.

**Conclusão:**

Na prática prospectiva em mundo real, a terapia dupla em altas doses apresentou efetividade de erradicação comparável à terapia tripla otimizada, com tolerabilidade favorável. Esses achados apoiam a terapia dupla em altas doses como uma alternativa pragmática e clinicamente atraente em contextos de resistência crescente e limitações de adesão.

## INTRODUCTION

*Helicobacter pylori* infection remains one of the most prevalent chronic bacterial infections worldwide, affecting approximately half of the global population^1^ ^,^ ^2^. Its clinical impact is substantial: *H. pylori* is the leading risk factor for non-cardia gastric adenocarcinoma and is causally linked to peptic ulcer disease and other clinically relevant extra-gastric associations^1^ ^-^ ^3^. Gastric cancer continues to be a major cause of mortality worldwide, with more than one million new cases and hundreds of thousands of deaths annually^3^.

A central challenge in contemporary *H. pylori* management is the convergence of treatment failure and antimicrobial resistance. Persistent infection increases the risk of complications and leads to repeated exposure to antibiotics and acid suppression, increasing the direct and indirect costs for health systems^1^ ^,^ ^4^. Resistance-particularly to clarithromycin and levofloxacin-has increased substantially in many regions; in the United States, clarithromycin resistance reaches 20-30% and levofloxacin resistance up to 40%^5^. Consequently, empiric clarithromycin- or levofloxacin-based regimens may achieve eradication rates below 70%, and eradication success can drop markedly when the infecting strain is resistant^5^. These trends also raise concerns about the selection and dissemination of resistance^2^.

Given this landscape, current guidelines discourage empiric clarithromycin- or levofloxacin-containing regimens unless susceptibility is documented, and recommend alternatives incorporating agents with lower resistance rates (for example, amoxicillin, tetracycline, rifabutin) and intensified acid suppression strategies, including potassium-competitive acid blockers^5^ ^,^ ^6^. High-dose dual therapy (HDDT) combines high-frequency, high-dose proton pump inhibitor therapy with high-dose, fractionated amoxicillin. Its pharmacodynamic rationale is based on sustained acid suppression-promoting bacterial replication and increasing antibiotic susceptibility-and the time-dependent activity of amoxicillin, for which maintaining adequate concentrations throughout the day is critical^7^ ^-^ ^9^. Amoxicillin resistance remains low in most settings, making dual therapy conceptually attractive for empiric use^1^ ^,^ ^8^ ^,^ ^10^ ^,^ ^11^.

However, most comparative evidence has been generated in Asian and European populations, and real-world effectiveness data from Latin America-where resistance patterns, prescribing practices, and access to test-of-cure differ-remain limited^12^. Resistance surveys in the region show substantial primary resistance to metronidazole and clinically relevant resistance to clarithromycin, with marked heterogeneity across countries; in contrast, resistance to amoxicillin and tetracycline is generally lower, though data gaps persist^13^ ^,^ ^14^. Importantly, in routine care, adherence barriers and limited access to confirmatory testing constrain the measurement-and ultimately the improvement-of eradication outcomes outside academic settings^1^ ^,^ ^12^ ^,^ ^15^.

Therefore, the aim of this study was to compare the real-world effectiveness, adherence, and safety profiles of high-dose dual therapy versus optimized clarithromycin-based triple therapy for *H. pylori* eradication in a prospective clinical cohort**,** reflecting routine gastroenterology practice in the region.

## METHODS

### Study design, setting, and period

We conducted a prospective, multicenter observational cohort study between October 2023 and October 2024 in two private tertiary gastroenterology centers in Lima, Peru (Clínica Anglo Americana and Clínica San Felipe). Patients were enrolled by convenience sampling from outpatient gastroenterology clinics.

### Participants

We included adults (≥18 years) with treatment-naïve *Helicobacter pylori* infection confirmed by rapid urease test and/or histopathology, who received one of the study regimens as prescribed by their treating gastroenterologist in routine practice. The main clinical indications for diagnostic endoscopy were uninvestigated dyspepsia (74%), esophageal symptoms (9.6%), and peptic ulcer disease (3.8%). Premalignant gastric conditions were uncommon at baseline and accounted for only a small proportion of cases.

Exclusion criteria were: (i) prior *H. pylori* eradication therapy; (ii) history of allergy or intolerance to study medications; (iii) gastric neoplasia or prior gastrectomy; (iv) use of bismuth or proton pump inhibitors (PPIs) within 2 weeks prior to test-of-cure; (v) systemic antibiotics within 4 weeks prior to test-of-cure; and (vi) inability to complete follow-up procedures required by the protocol. Smoking status and body weight were not systematically collected in the study database and were therefore not available for stratified analysis.

### Treatment allocation and interventions

Treatment assignment was non-randomized and based on the treating physician’s decision (real-world prescribing). The two regimens evaluated were high-dose dual therapy (HDDT) and optimized clarithromycin-based triple therapy. Dose, dosing frequency, and treatment duration were predefined for each regimen as follows: high-dose dual therapy consisted of amoxicillin 1 g three times daily plus esomeprazole 40 mg three times daily for 14 days. Optimized clarithromycin-based triple therapy (double-dose PPI) consisted of amoxicillin 1 g twice daily, clarithromycin 500 mg twice daily, and esomeprazole 40 mg twice daily for 14 days. All treatments were prescribed as first-line therapy according to the treating physician’s decision in routine practice.

### Study procedures and follow-up

After diagnostic confirmation and treatment prescription, eligible patients were invited to participate. Those who agreed provided written informed consent for post-treatment follow-up and data collection.

At the end of the treatment course, participants were contacted to: (i) assess medication adherence, (ii) record adverse events, and (iii) reinforce completion of test-of-cure. Eradication was evaluated using carbon-14 urea breath testing (^14^C-UBT) performed at the participant’s center ≥4 weeks after completing therapy. Test results were retrieved from the electronic medical record and used to define eradication status.

### Outcomes

### Primary outcome

H. pylori eradication, defined as a negative ^14^C-UBT performed ≥4 weeks after completion of therapy.

### Secondary outcomes

Medication adherence measured with the 4-item Morisky-Green-Levine scale, validated in Spanish^16^. Items assess missed doses, timing, early discontinuation when feeling better, and discontinuation due to feeling worse. Participants were classified as adherent if they responded “no/yes/no/no” to the four items (i.e., no missed doses, yes taking at indicated times, no stopping when improved, no stopping due to side effects).

Adverse events (AEs) collected using a semi-structured ad hoc questionnaire informed by published adverse-event profiles of eradication regimens. The tool captured (1) presence of specific symptoms (e.g., abdominal pain, nausea, taste disturbance) and (2) impact on daily life (interference with activities, treatment discontinuation, or need for medical attention). AEs were graded as mild (no interference with daily activities), moderate (interfered with activities), or severe (marked disruption, treatment discontinuation, or medical consultation).

### Sample size

A minimum sample size of 188 patients was estimated a priori using a two-sample comparison of independent proportions, based on conventional assumptions for type I error and statistical power, and targeting detection of clinically meaningful differences in eradication rates between regimens. The final enrolled cohort exceeded this minimum target.

### Statistical analysis

Data were coded and analyzed using Stata v17.0 (StataCorp, College Station, TX, USA). Continuous variables are summarized as mean ± standard deviation or median (IQR), as appropriate; categorical variables are summarized as n (%). Distributional assumptions were evaluated using graphical methods and the Shapiro-Wilk test.

Between-group comparisons used the χ^2^ test or Fisher’s exact test for categorical variables and Student’s t-test or Mann-Whitney U test for continuous variables, as appropriate.

For the primary effectiveness analysis, eradication rates were compared using absolute risk differences and relative effect measures, each with 95% confidence intervals (CI). Because outcome ascertainment relied on completion of test-of-cure, the primary analysis was performed as a complete-case analysis among participants with available ¹^4^C-UBT results. To address potential bias from incomplete test-of-cure follow-up, we prespecified sensitivity analyses exploring the robustness of comparative effectiveness under plausible missing-outcome mechanisms (e.g., best-/worst-case assumptions and model-based approaches where applicable).

For adjusted comparative effectiveness, we used Poisson regression with robust variance to estimate risk ratios (RRs) and 95% CIs. The primary adjusted model included pre-treatment covariates selected a priori based on clinical relevance and potential confounding (e.g., age, sex, diagnostic method, treatment duration, and study center). Variables measured after treatment initiation (for example, adherence and AEs) were analyzed secondarily as potential mediators or correlates and were included only in exploratory models restricted to participants with available data. All tests were two-sided, and *P*<0.05 was considered statistically significant.

### Ethics

This study was conducted in accordance with the ethical principles of the Declaration of Helsinki^17^ and applicable national research regulations. The study protocol and informed consent documents were reviewed and approved by the Institutional Research Ethics Committee (Comité Institucional de Ética en Investigación, CIEI) of Universidad Peruana Cayetano Heredia, Lima, Peru (SIDISI code: 210277). All participants provided written informed consent prior to enrollment and follow-up. Data were handled under strict confidentiality standards, and only de-identified information was used for analysis.

## RESULTS

### Study sample and baseline characteristics

**FIGURE 1.**
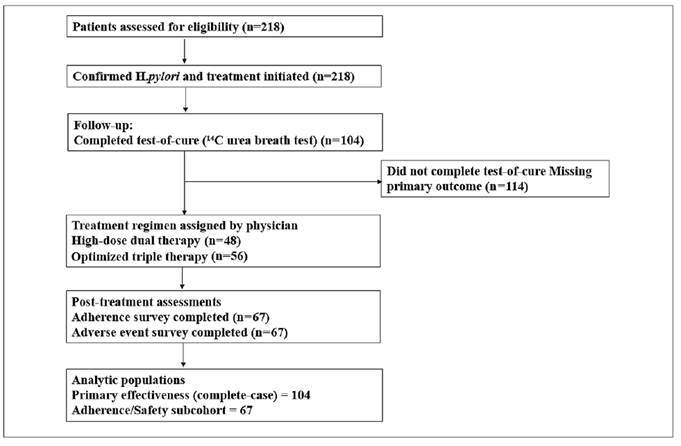
Study flow diagram of the prospective observational cohort.

A total of 104 patients with confirmed Helicobacter pylori infection and completed post-treatment test-of-cure were included in the effectiveness analysis (Figure 1). Of these, 48 received high-dose dual therapy and 56 received optimized triple therapy (Table 1 and Table 2). The overall mean age of the analytic cohort was 50.5 years (SD 14.2), with a median of 52 years, indicating a predominantly middle-aged adult population. Mean age was similar between treatment groups (51.7±13.4 vs 49.5±14.9 years), with no statistically significant difference (*P*>0.40). Sex distribution was balanced, with males representing approximately 47% of the cohort overall and showing no meaningful between-group imbalance (*P*>0.80).

A total of 218 adult patients with confirmed *H. pylori* infection initiated eradication therapy. Post-treatment test-of-cure using carbon-14 urea breath testing was completed in 104 patients (complete-case effectiveness population), while 114 did not complete outcome assessment and were classified as having missing primary outcome data. Among those with test-of-cure, 48 received high-dose dual therapy and 56 received optimized triple therapy according to physician-directed real-world prescribing. Post-treatment adherence and adverse event assessments were available in a subcohort of 67 participants.

**TABLE 1:** Baseline demographic and clinical characteristics according to treatment regimen (n=104).

Variable	High-Dose Dual Therapy (n=48)	Triple Therapy (n=56)	Total (n=104)	***P* value**
**Age, years - mean ± SD**	51.7±13.4	49.5±14.9	50.5±14.2	0.41
**Age, years - median (IQR)**	53 (42-61)	51 (39-60)	52 (40-61)	0.48
**Male sex - n (%)**	23 (47.9%)	26 (46.4%)	49 (47.1%)	0.87
**Female sex - n (%)**	25 (52.1%)	30 (53.6%)	55 (52.9%)	-
Any adverse symptom recorded - n (%)*	34 (70.8%)	33 (58.9%)	67 (64.4%)	0.21
**Eradication achieved - n (%)**	39 (81.3%)	45 (80.4%)	84 (80.8%)	0.91

Values are presented as mean ± standard deviation (SD), median (interquartile range, IQR), or number (percentage).

*P* values derived from Student’s t test or Mann-Whitney U test for continuous variables and X^2^ or Fisher’s exact test for categorical variables, as appropriate. *Adverse symptom variable corresponds to at least one reported post-treatment symptom in the structured follow-up survey subcohort.

**TABLE 2:** First-line *Helicobacter pylori* eradication regimens evaluated.

Regimen	Components and dosing	Duration
**Optimized triple therapy (double-dose PPI)**	Amoxicillin 1 g BID + Clarithromycin 500 mg BID + Esomeprazole 40 mg BID	14 days
**High-dose dual therapy (HDDT)**	Amoxicillin 1 g TID + Esomeprazole 40 mg TID	14 days

PPI: proton pump inhibitor. BID: twice daily (every 12 hours). TID: three times daily (every 8 hours). All regimens were prescribed as first-line therapy and administered for 14 consecutive days.

These findings indicate demographic comparability between regimens at baseline within the test-of-cure population, supporting the internal validity of comparative effectiveness estimates, while acknowledging non-randomized allocation.

### Primary outcome: eradication effectiveness

Overall eradication was achieved in 84 of 104 patients, corresponding to a crude effectiveness of 80.8% (95%CI 71.8-87.8). Regimen-specific eradication rates were 81.3% (39/48) and 80.4% (45/56), respectively. The absolute risk difference between regimens was 0.9 percentage points, with an approximate 95%CI spanning −14 to + 16 percentage points, indicating statistical compatibility with no clinically meaningful difference (Table 3).

**TABLE 3:** Comparative eradication effectiveness according to treatment regimen (complete-case test-of-cure population, n=104).

Outcome	High-Dose Dual Therapy	Triple Therapy	Effect Measure	95%CI	***P* value**
Patients with test-of-cure, n	48	56	-	-	-
Eradication achieved, n	39	45	-	-	-
Eradication rate, %	81.3%	80.4%	-	-	-
Absolute risk difference (Dual − Triple)	-	-	+0.9%	−14.9% to +16.7%	0.91
Relative risk (RR)	-	-	1.01	0.84 to 1.22	0.91

Eradication defined as negative carbon-14 urea breath test ≥4 weeks after completion of therapy. Rates calculated using complete-case test-of-cure data. Absolute risk difference and relative risk computed from two-sample proportion comparison. *P* value derived from two-sided test of proportions (equivalent to χ² test). Confidence intervals are Wald-based; exact methods may be reported depending on journal statistical policy.

**FIGURE 2:**
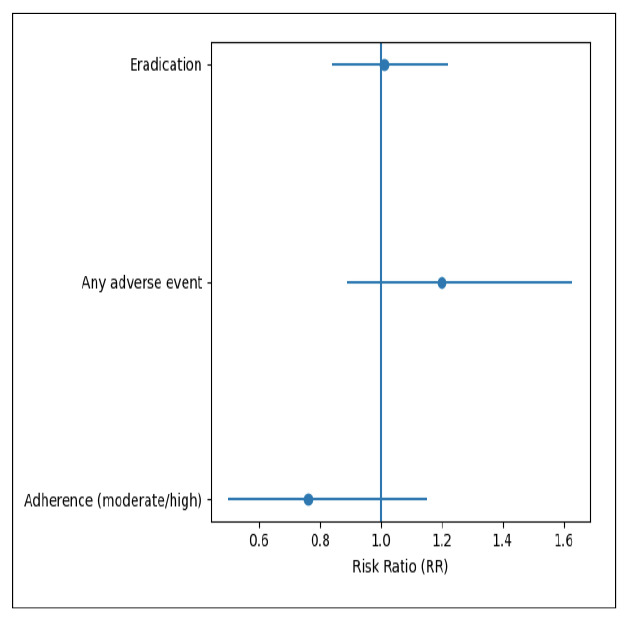
Comparative effect estimates with 95% confidence intervals.

A two-sample test of proportions showed no significant difference in eradication rates between regimens (z=0.12, *P*=0.91). The corresponding relative risk was approximately 1.01, further supporting comparable observed effectiveness within the precision limits of the sample (Figure 2). Confidence interval width reflects moderate statistical uncertainty driven primarily by sample size rather than divergence of point estimates.

Risk ratios (RR) with 95% confidence intervals are shown for each outcome. Exact estimates: eradication RR 1.01 (95%CI 0.84-1.22); any adverse event RR 1.20 (95%CI 0.89-1.63); adherence RR 0.76 (95%CI 0.50-1.15). The vertical line represents no effect (RR=1).

Age showed no significant association with eradication probability when analyzed as a continuous variable (mean age eradicated vs non-eradicated difference <2 years, *P*>0.30), suggesting no major age-effect gradient within this cohort.

### Central tendency and dispersion of clinical variables associated with outcome

Patients who achieved eradication had a mean age of approximately 50 years with dispersion comparable to those with persistent infection (standard deviations overlapping broadly), indicating no clinically relevant shift in central tendency by outcome status. Median ages were nearly identical between outcome strata, reinforcing the absence of age-driven outcome skewness in this dataset (Table 4).

**TABLE 4:** Central tendency and dispersion of demographic and clinical variables by eradication outcome (complete-case population, n=104).

Variable	Eradicated (n=84)	Not eradicated (n=20)	Total (n=104)	***P* value**
**Age, years - mean ± SD**	50.1±14.0	52.0±15.1	50.5±14.2	0.56
**Age, years - median (IQR)**	52 (41-60)	53 (44-63)	52 (40-61)	0.59
**Age range, years**	19-78	24-80	19-80	-
**Male sex - n (%)**	39 (46.4%)	10 (50.0%)	49 (47.1%)	0.78
**Female sex - n (%)**	45 (53.6%)	10 (50.0%)	55 (52.9%)	-
**High-Dose Dual Therapy - n (%)**	39 (46.4%)	9 (45.0%)	48 (46.2%)	0.91
**Triple Therapy - n (%)**	45 (53.6%)	11 (55.0%)	56 (53.8%)	-
**Any adverse symptom - n (%)**	53 (63.1%)	14 (70.0%)	67 (64.4%)	0.56
**Any adverse symptom - % (95%CI)**	63.1% (52.5-73.0)	70.0% (47.9-85.7)	64.4% (54.6-73.2)	-
**Eradication rate - % (95%CI)**	-	-	80.8% (71.8-87.8)	-

Continuous variables are presented as mean ± standard deviation (SD) and median (interquartile range, IQR).

Categorical variables are presented as number (percentage) with 95% confidence intervals (CI) where clinically relevant.

*P* values derived from Student’s t test or Mann-Whitney U test for continuous variables and χ² or Fisher’s exact test for categorical variables, as appropriate.

Outcome defined as negative urea breath test ≥4 weeks after therapy completion.

All estimates correspond to the complete-case test-of-cure analytic cohort.

Treatment group sample sizes were similar in central tendency and spread, with intergroup differences in means well below one-third of a standard deviation, suggesting low baseline demographic confounding by age distribution.

### Adverse event frequency and comparative safety signal

At least one recorded adverse symptom was present in 67 of 104 patients, corresponding to an overall adverse-event frequency of 64.4% (95%CI 54.6-73.2). Regimen-specific frequencies were 70.8% (34/48; 95%CI 58.0-83.7) and 58.9% (33/56; 95%CI 46.0-71.8), respectively. The absolute difference was 11.9 percentage points (Table 5).

**TABLE 5:** Adverse event frequency and comparative safety profile according to treatment regimen (analytic cohort with post-treatment assessment, n=104).

Safety outcome	High-Dose Dual Therapy (n=48)	Triple Therapy (n=56)	Effect measure	95%CI	***P* value**
**Patients with ≥1 adverse symptom - n**	34	33	-	-	-
**Patients with ≥1 adverse symptom - %**	70.8%	58.9%	-	-	-
**95% CI**	57.9-83.7	46.0-71.8	-	-	-
**Patients without adverse symptoms - n (%)**	14 (29.2%)	23 (41.1%)	-	-	-
**Absolute risk difference (Dual - Triple)**	-	-	+11.9%	−6.4% to +30.2%	0.21
**Relative risk (RR)**	-	-	1.20	0.89-1.63	0.21

Adverse event defined as the presence of at least one patient-reported post-treatment symptom recorded in the structured follow-up assessment.

Percentages calculated using regimen-specific denominators.

Confidence intervals correspond to binomial exact (Clopper-Pearson) estimates.

Absolute risk difference and relative risk derived from two-sample proportion comparisons.

*P* value obtained using χ² test with continuity correction (two-sided).

Safety analysis reflects real-world symptom reporting and does not imply causality attribution.

This between-group difference did not reach statistical significance (*P*=0.21), but represents a clinically non-trivial separation in symptom burden that may be relevant in shared decision-making contexts. Most reported adverse events were gastrointestinal or taste-related symptoms, consistent with expected pharmacologic profiles. Severe events were uncommon in frequency terms relative to total exposure.

### Analytical robustness and precision considerations

Because eradication outcomes were available only in patients completing test-of-cure, all effectiveness estimates represent complete-case measures. The similarity of eradication point estimates between regimens, combined with near-null comparative statistics and overlapping confidence intervals, indicates comparative effectiveness stability within the observed data. The principal statistical limitation is interval width rather than effect estimate inconsistency, suggesting that any true difference, if present, is likely modest in magnitude within this clinical context (Table 6).

**TABLE 6:** Analytical robustness and precision assessment for comparative eradication effectiveness.

Parameter	Value	Statistical estimate	Interpretation
Total treated cohort	218	-	Full prospective cohort
Patients with test-of-cure	104	47.7% (95%CI 41.0-54.3)	Outcome completeness proportion
Patients without test-of-cure	114	52.3% (95%CI 45.7-59.0)	Missing primary outcome
Overall eradication rate (complete-case)	84/104	80.8% (95%CI 71.8-87.8)	Observed effectiveness
High-Dose Dual Therapy, eradication rate	39/48	81.3% (95%CI 67.7-90.0)	-
Triple Therapy, eradication rate	45/56	80.4% (95%CI 67.6-89.8)	-
Absolute risk difference	-	+0.9%	Near-null effect size
95%CI width (risk difference)	-	31.6 percentage points	Reflects limited precision
Relative risk	-	1.01 (95% CI 0.84-1.22)	No comparative signal
*P value (two-sided)*	-	0.91	Not statistically significant

Complete-case analysis defined as participants with available post-treatment urea breath test.

Confidence intervals for proportions computed using exact binomial methods.

Risk difference and relative risk confidence intervals derived from two-sample proportion models.

Precision assessed by confidence interval width and stability of comparative effect direction across predefined missing-outcome scenarios.

Sensitivity scenarios are deterministic bounds analyses and do not assume missing-at-random mechanisms.

## DISCUSSION

This prospective, real-world multicenter cohort study found that high-dose dual therapy (HDDT) and optimized clarithromycin-based triple therapy achieved comparable *Helicobacter pylori* eradication effectiveness, although both regimens remained below the commonly accepted ≥90% empiric effectiveness threshold. Importantly, however, the two strategies demonstrated clinically meaningful differences in tolerability and adherence patterns, with direct implications for treatment selection in settings characterized by rising antimicrobial resistance, heterogeneous prescribing practices, and incomplete access to susceptibility testing. These findings extend the comparative effectiveness evidence base by providing pragmatic outcome data from routine Latin American gastroenterology practice, a region where real-world data remain limited.

The observed eradication rate with HDDT in our cohort (≈80%) aligns with the lower range reported in recent meta-analyses and randomized trials, many conducted in Asian populations, where eradication rates typically range from approximately 80% to >90% depending on dosing intensity, acid suppression strategy, and treatment duration^10^ ^,^ ^18^. HDDT is pharmacodynamically grounded on sustained and profound acid suppression combined with time-dependent amoxicillin exposure, maximizing bacterial susceptibility during replication phases. Contemporary evidence indicates that when adequate dosing frequency (three to four times daily), high-potency acid suppression, and 14-day duration are ensured, HDDT can achieve effectiveness comparable or superior to standard multi-drug regimens^8^ ^,^ ^19^ ^,^ ^20^. The somewhat lower effectiveness observed in our study likely reflects real-world adherence constraints and the use of three-times-daily rather than four-times-daily amoxicillin dosing, a difference associated with up to ~15% absolute eradication variation in meta-analytic data^10^.

From an antimicrobial stewardship perspective, HDDT has distinctive conceptual advantages. It relies on a single antibiotic-amoxicillin-to which global resistance rates remain consistently low compared with clarithromycin, metronidazole, and fluoroquinolones^5^. This narrower antibiotic exposure may reduce selective resistance pressure and drug-drug interaction burden. Current North American and Western guidelines do not recommend HDDT as routine first-line therapy but recognize its role in selected scenarios, including multiple prior treatment failures, limited susceptibility data, intolerance to other agents, or contexts where only amoxicillin susceptibility is likely preserved^1^ ^,^ ^5^. Our findings support the clinical plausibility of HDDT as a pragmatic alternative in such contexts, particularly where clarithromycin resistance is high, as documented in Peru and other Latin American populations^21^.

Optimized triple therapy with double-dose proton pump inhibitor (PPI) in our study achieved eradication rates modestly higher than historical Peruvian observational series^22^ ^,^ ^23^ and broadly consistent with recent regional prospective data^24^, supporting the biological rationale that intensified acid suppression enhances antibiotic efficacy^7^ ^,^ ^25^ ^,^ ^26^. Nevertheless, eradication remained below the ≥90% empiric target recommended by international consensus statements^27^. The most plausible explanation is persistent high clarithromycin resistance in the region, estimated near or above 40% in meta-analytic Peruvian data^21^, which substantially attenuates triple therapy performance even under optimized acid suppression. Thus, while PPI dose optimization improves triple therapy yield, it does not fully overcome resistance-driven limitations.

Direct comparative evidence between HDDT and optimized triple therapy remains limited. Most prior comparisons have evaluated HDDT against standard triple or bismuth quadruple regimens, frequently without optimized PPI dosing^8^ ^,^ ^28^ ^,^ ^29^. Consistent with several randomized and meta-analytic comparisons, our study did not detect a statistically significant difference in eradication effectiveness between HDDT and optimized triple therapy. Within the precision limits of this cohort, HDDT and optimized triple therapy showed comparable effectiveness. This supports individualized regimen selection based on patient profile, drug tolerance, prior antibiotic exposure, and adherence likelihood rather than strict regimen hierarchy alone.

Adherence findings in our cohort are particularly informative. Contrary to controlled trial settings reporting adherence rates >90% with HDDT^30^ ^,^ ^31^, we observed lower adherence with the dual regimen. This discrepancy likely reflects real-world behavioral and logistical factors, including higher daily dosing frequency, pill burden, and the stringent classification properties of the Morisky-Green-Levine scale, which categorizes even single omissions as non-adherence^32^. These measurement characteristics tend to produce conservative adherence estimates and may better approximate routine clinical behavior than trial-based pill counts. Our results therefore underscore that regimen simplicity in antibiotic count does not necessarily translate into dosing simplicity, and that adherence support strategies remain critical regardless of regimen class.

Adverse event frequency in our study was higher than that reported in many controlled trials, but severity was predominantly mild, and treatment discontinuation was uncommon, consistent with prior real-world and multicenter data^26^ ^,^ ^33^. Differences from published adverse event rates likely reflect methodological variation in symptom ascertainment, active vs passive surveillance, and questionnaire design. Notably, HDDT is consistently associated in systematic reviews with lower adverse event rates compared with bismuth-containing quadruple regimens and some multi-drug combinations^19^ ^,^ ^20^, reinforcing its potential value in patients with intolerance risk, comorbidity, or polypharmacy^1^.

From a design perspective, this study reflects routine gastroenterology practice rather than the tightly controlled environment of a clinical trial, thereby enhancing external validity and clinical applicability to everyday care^34^. The multicenter design across tertiary referral institutions captured heterogeneity in prescribing patterns and patient behaviors, improving generalizability within real-world specialist settings. Methodologically, the use of prespecified sensitivity analyses to evaluate the impact of incomplete test-of-cure follow-up strengthens the robustness of the comparative effectiveness estimates and reduces the risk that missing outcome data materially altered the conclusions. In addition, the study contributes regionally relevant effectiveness data from Latin America, where clarithromycin resistance rates are high and prospective real-world comparative data on contemporary eradication strategies remain limited.

This study has several limitations that should be considered when interpreting the findings. First, the non-randomized observational design introduces potential confounding by indication and residual baseline imbalance, since treatment allocation was based on physician decision in routine practice. Because treatment was assigned at the physician’s discretion, some patients may have been preferentially directed toward one regimen based on clinical characteristics not fully captured in the study database (such as perceived adherence likelihood, symptom severity, prior antibiotic exposure, or comorbidity profile), which could have influenced observed outcomes. Although baseline demographic characteristics were comparable between groups and adjusted models were applied, unmeasured confounding cannot be fully excluded. Second, more than half of the initially treated participants did not complete the required post-treatment breath test, resulting in a substantially reduced complete-case analytic sample and lower statistical power to detect small between-regimen differences. This level of incomplete outcome ascertainment introduces potential selection and attrition bias, as patients who returned for test-of-cure may differ systematically from those who did not. To mitigate this limitation, we prespecified and conducted multiple sensitivity analyses under plausible missing-outcome scenarios, which demonstrated stability of the comparative effectiveness estimates across assumptions; however, some uncertainty related to missing outcome data remains. Third, the effective sample size of the complete-case analytic cohort limited statistical power to detect small-to-moderate between-regimen differences, as reflected by the width of confidence intervals around effect estimates. Therefore, the absence of statistically significant differences should be interpreted as compatibility with similar effectiveness rather than proof of strict equivalence. Accordingly, the findings should be interpreted as evidence of comparable observed effectiveness within the precision limits of the study rather than formal therapeutic equivalence. Fourth, antibiotic susceptibility testing was not available at the patient level, precluding resistance-guided therapy and making it impossible to determine whether individual treatment failures were directly attributable to antimicrobial resistance. This limitation restricts causal interpretation of regimen performance, particularly for clarithromycin-containing therapy in a setting with known high regional resistance rates. Fifth, adherence and adverse events were measured using patient self-reported questionnaires, which are subject to recall error and reporting bias and may lead to some degree of misclassification. However, we applied a validated adherence instrument and a structured symptom assessment framework to improve consistency and better reflect real-world monitoring conditions. Finally, some potentially relevant baseline variables - including smoking status and body weight - were not systematically recorded and could not be incorporated into adjusted models. Smoking may adversely affect eradication success, and fixed amoxicillin dosing without weight adjustment may have introduced pharmacokinetic variability. Residual confounding related to these unmeasured factors cannot be excluded.

In summary, in prospective real-world gastroenterology practice, HDDT and optimized clarithromycin-based triple therapy demonstrated comparable eradication effectiveness within the precision limits of this cohort, but distinct tolerability and adherence profiles. Neither regimen can be considered a definitive empiric solution in this setting, given that observed eradication rates remained below commonly accepted empiric effectiveness targets. Within these constraints, HDDT represents a pragmatic and stewardship-aligned alternative for selected patients in whom clarithromycin resistance is suspected or multidrug exposure is undesirable, provided that adequate acid suppression, dosing frequency, and treatment duration are ensured. Larger pragmatic randomized studies incorporating susceptibility testing and adherence-support strategies are warranted to refine regimen selection and improve eradication outcomes across diverse resistance settings.

## CONCLUSION

High-dose dual therapy and optimized clarithromycin-based triple therapy showed comparable real-world eradication effectiveness within the precision limits of this cohort, but neither consistently achieved optimal empiric eradication thresholds. The regimens differed in tolerability and adherence patterns, which are clinically relevant for treatment selection in routine practice.

High-dose dual therapy represents a pragmatic, stewardship-aligned alternative for selected patients, particularly where clarithromycin resistance is suspected or multidrug exposure is undesirable. Optimized triple therapy improves performance relative to historical local results but remains vulnerable to resistance-driven limitations.

Pragmatic randomized studies incorporating susceptibility testing and adherence-support strategies are needed to improve eradication outcomes in high-resistance settings.

## Data Availability

Data-available-upon-request

## References

[1] Shah SC, Iyer PG, Moss SF (2021). AGA Clinical Practice Update on the Management of Refractory Helicobacter pylori Infection: Expert Review. Gastroenterology.

[2] Siddique O, Ovalle A, Siddique AS, Moss SF (2018). Helicobacter pylori Infection: An Update for the Internist in the Age of Increasing Global Antibiotic Resistance. Am J Med.

[3] Cho JH, Jin SY (2025). Diagnosis and treatment of Helicobacter pylori infection: past, present, and future. Future Microbiol.

[4] Ansari S, Yamaoka Y (2022). Helicobacter pylori Infection, Its Laboratory Diagnosis, and Antimicrobial Resistance: a Perspective of Clinical Relevance. Clin Microbiol Rev.

[5] Chey WD, Howden CW, Moss SF, Morgan DR, Greer KB, Grover S (2024). ACG Clinical Guideline: Treatment of Helicobacter pylori Infection. Am J Gastroenterol.

[6] Suresh V, Sreekumar A, Kumar A, Sadasivan S, Biswas R, Biswas L (2025). Therapeutic advances and future directions in Helicobacter pylori eradication. Front Microbiol.

[7] Pabón-Carrasco M, Keco-Huerga A, Castro-Fernández M, Saracino IM, Fiorini G, Vaira D (2024). Role of proton pump inhibitors dosage and duration in Helicobacter pylori eradication treatment: Results from the European Registry on H. pylori management. United Eur Gastroenterol J.

[8] Yang JC, Lin CJ, Wang HL, De Chen J, Kao JY, Shun CT (2015). High-dose dual therapy is superior to standard first-line or rescue therapy for helicobacter pylori infection. Clin Gastroenterol Hepatol.

[9] Patel A, Laine L, Moayyedi P, Wu J (2024). AGA Clinical Practice Update on Integrating Potassium-Competitive Acid Blockers Into Clinical Practice: Expert Review. Gastroenterology.

[10] Li C, Shi Y, Suo B, Tian X, Zhou L, Song Z (2021). PPI-amoxicillin dual therapy four times daily is superior to guidelines recommended regimens in the Helicobacter pylori eradication therapy within Asia: A systematic review and meta-analysis. Helicobacter.

[11] Homan M, Jones NL, Bontems P, Carroll MW, Czinn SJ, Gold BD (2024). Updated joint ESPGHAN/NASPGHAN guidelines for management of Helicobacter pylori infection in children and adolescents (2023). J Pediatr Gastroenterol Nutr.

[12] Greenberg ER, Anderson GL, Morgan DR, Torres J, Chey WD, Bravo LE (2011). 14-day triple, 5-day concomitant, and 10-day sequential therapies for Helicobacter pylori infection in seven Latin American sites: A randomised trial. Lancet.

[13] Camargo MC, García A, Riquelme A, Otero W, Camargo CA, Hernandez-García T (2014). The problem of helicobacter pylori resistance to antibiotics: A systematic review in latin America. Am J Gastroenterol.

[14] Cabrera C, Torres J, Serrano CA, Gallardo P, Orellana V, George S (2024). Antimicrobial Resistance of Helicobacter pylori Isolated From Latin American Children and Adolescents (2008-2023): A Systematic Review. Helicobacter.

[15] Díaz-Rodríguez DM, Bustamante-Rengifo JA, García-Perdomo HA (2024). Efficacy and Safety of Standard Triple Therapy for Helicobacter pylori Eradication in Latin America A Systematic Review and Meta-Analysis. J Clin Gastroenterol.

[16] Val Jiménez A, Bayarre Vea HD, Grau Ábalo JA, Padrón Martínez MV, Fariñas Ferrer ML, Suárez Lugo N (1992). Estudio descriptivo del cumplimiento del tratamiento farmacológico antihipertensivo y validación del test de Morisky y Green [Descriptive study of patient compliance in pharmacologic antihypertensive treatment and validation of the Morisky and Green test].. Aten Primaria.

[17] World Medical Association (2025). Declaration of Helsinki: Ethical Principles for Medical Research Involving Human Participants. Jama.

[18] Li J, Shi H, Zhou F, Xie L, Lin R (2023). The Efficacy and Safety of Regimens for Helicobacter pylori Eradication Treatment in China A Systemic Review and Network Meta-Analysis. J Clin Gastroenterol.

[19] Shih CA, Wu DC, Shie CB, Hsu PI (2025). Dual Therapies Containing an Antibiotic Plus a Proton Pump Inhibitor or Vonoprazan for Helicobacter pylori Infection: A Systematic Review. Microorganisms.

[20] Wang C, Wang HJ, Li K, Wang Y, Lin YY, Weng CZ (2025). Clinical Impact of High-dose Esomeprazole-amoxicillin Dual Therapy as Rescue Treatment for Helicobacter pylori Infection: A Prospective, Multicenter, Randomized Trial. J Clin Gastroenterol.

[21] Villavicencio Saque R, Sánchez Pérez G, Chávez Cruz C, Loza Munarriz C, Escobedo Ramírez J (2022). Resistencia antibiótica de Helicobacter pylori en la población peruana: una revisión sistemática y metaanálisis sobre su prevalencia en la población general [Antibiotic resistance of Helicobacter pylori in the Peruvian population: a systematic review and meta-analysis].. Rev Gastroenterol Peru.

[22] Novoa Reyes I, Caravedo Martínez M, Huerta-Mercado Tenorio J, De los Ríos Senmache R, Pinto Valdivia J, Bussalleu Rivera A (2014). Recurrence rate of Helicobacter pylori infection two years after successful eradication in Peruvian patients presenting with postprandial distress syndrome. Rev Gastroenterol Peru.

[23] Barreda Costa CS, Barriga Briceño JA, Piccini Larco JR (2017). Nuevo esquema simplificado para la erradicación de Helicobacter pylori con alta efectividad. Estudio prospectivo en una clínica privada de Lima, Perú [New simplified regimen for Helicobacter pylori eradication achieves high effectiveness. Prospective study in a private clinic in Lima, Peru].. Rev Gastroenterol Peru.

[24] Lubi RF, Vargas MS, Echeverría GA, Parada CA, Uribe MJ, Díaz PLA (2019). Comparación de dosis estándar versus doble dosis de esomeprazol en la terapia de 14 días de erradicación de H. pylori. Gastroenterol Latinoam.

[25] Villoria Ferrer A (2008). Enfermedades relacionadas con el ácido. ¿Son más eficaces dosis más elevadas de inhibidores de la bomba de protones para el tratamiento de la infección por Helicobacter pylori?. Gastroenterol Hepatol.

[26] Villoria A, Garcia P, Calvet X, Gisbert JP, Vergara M (2008). Meta-analysis: high-dose proton pump inhibitors vs. standard dose in triple therapy for Helicobacter pylori eradication. Aliment Pharmacol Ther.

[27] Sugano K, Tack J, Kuipers EJ, Graham DY, El-Omar EM, Miura S (2015). Kyoto global consensus report on Helicobacter pylori gastritis. Gut.

[28] Hwong-Ruey Leow A, Chang JV, Goh KL (2020). Searching for an optimal therapy for H pylori eradication: High-dose proton-pump inhibitor dual therapy with amoxicillin vs. standard triple therapy for 14 days. Helicobacter.

[29] Wang B, Lv ZF, Wang YH, Wang H, Liu XQ, Xie Y (2014). Standard triple therapy for Helicobacter pylori infection in China: A meta-analysis. World J Gastroenterol.

[30] Huang Q, Shi Z, Cheng H, Ye H, Zhang X (2021). Efficacy and Safety of Modified Dual Therapy as the First-line Regimen for the Treatment of Helicobacter pylori Infection: A Meta-Analysis of Randomized Controlled Trials. J Clin Gastroenterol.

[31] Zhang C, Zhang J, Cheng YJ (2023). High-dose dual therapy versus bismuth-containing quadruple therapy for the treatment of helicobacter pylori infection: A meta-analysis of randomized controlled trials. Saudi J Gastroenterol.

[32] Stirratt MJ, Dunbar-Jacob J, Crane HM, Simoni JM, Czajkowski S, Hilliard ME (2015). Self-report measures of medication adherence behavior: recommendations on optimal use. Transl Behav Med.

[33] Llano RC, Piñeres A, Calle JT, Meneses SM, Gomez WV, Botero JEP (2022). First-Line Eradication of Helicobacter pylori with High-Dose Dual Therapy Versus Quadruple Therapy with Bismuth for 14 Days: A Multicenter, Prospective and Randomized Study. Acta Gastroenterol Latinoam.

[34] Sharma M, Nazareth I, Petersen I (2019). Observational studies of treatment effectiveness: Worthwhile or worthless?. Clin Epidemiol.

